# Genetic polymorphisms of *TLR3 *are associated with Nasopharyngeal carcinoma risk in Cantonese population

**DOI:** 10.1186/1471-2407-7-194

**Published:** 2007-10-17

**Authors:** Jun-Fang He, Wei-Hua Jia, Qin Fan, Xin-Xi Zhou, Hai-De Qin, Yin Yao Shugart, Yi-Xin Zeng

**Affiliations:** 1 State Key Laboratory of Oncology in Southern China; 2 Department of Experimental Research, Sun Yat-sen University, Cancer Center, Guangzhou, China; 3 Department of Epidemiology, Johns Hopkins Bloomberg School of Public Health, Baltimore, Maryland, USA

## Abstract

**Background:**

Nasopharyngeal carcinoma is endemic in Southern China, displays a strong relationship with genetic susceptibility and associates with Epstein-Barr virus infection. Toll-like receptor 3 (TLR3) plays an important role in the antivirus response. Therefore, we examined the association between *TLR3 *gene polymorphisms and NPC susceptibility.

**Methods:**

We performed a case-control study of 434 NPC cases and 512 healthy controls matched on age, sex and residence. Both cases and controls are of Cantonese origin from Southern China. Genetic variants in *TLR3 *were determined by polymerase chain reaction (PCR)-based DNA direct sequencing and four SNPs were genotyped in all samples.

**Results:**

Our results showed that allele C for SNP 829A/C increased NPC risk significantly ((p = 0.0068, OR = 1.49, 95%CI:1.10–2.00). When adjusted for age, gender and VCA-IgA antibody titers, the NPC risk was reduced significantly among individuals who carried the haplotype "ATCT" compared to those who carried the most common haplotype "ACCT" (p = 0.0054, OR = 0.028; 95% CI (0.002–0.341).

**Conclusion:**

The *TLR3 *polymorphisms may be relevant to NPC susceptibility in the Cantonese population, although the reduction in NPC risk is modest and the biological mechanism of the observed association merits further investigation.

## Background

Nasopharyngeal carcinoma (NPC) occurs sporadically in the West (with the age – standardized incidence rat (ASR) < 1/100,000), but is a leading form of tumor in Southern China (ASR = 30–50/100,000) and Southeast Asia (ASR = 9–12/100,000) [1,2]. The geographical pattern of incidence suggests a unique interaction of environmental and genetic factors. Although the etiology of NPC remains to be elucidated, genetic susceptibility [3-6], Epstein-Barr virus (EBV) infection association [7-11], environmental risk factors [12,13] and certain dietary factors [14,15] may all contribute to the development of NPC. EBV, a ubiquitous virus that infects more than 90% of the world's population by adulthood, is an important risk factor for the development of NPC; however, NPC occurs in only a small percentage of the EBV-infected population [16]. The absence of NPC in most healthy EBV carriers is reportedly due to the effective T cell-mediated immune control of the virus [17]. It is known that HLA class I-restricted cytotoxic T-lymphocytes (CTLs) play an important role in controlling EBV infections [2]. When the cells are infected with EBV, they express an array of EBV-associated antigens and these viral antigens which are targeted by EBV-specific CTLs. The responses of CTLs to EBV infection trigger a variety of inflammatory reactions that can kill the infected cells, while the lack of CTLs allows EBV-infected cells to survive and proliferate [18]. It has been shown that some EBV strains are able to escape immune surveillance in a certain group of the population [19]. Studies in NPC cell lines indicate that the tumor is capable of processing endogenously expressed EBV antigens for recognition by HLA class I-restricted CTLs, resulting in lysis of the malignant cells [20].

Toll-like receptors (TLRs) have emerged as a key component of the innate immune system that recognizes a wide variety of pathogen-associated molecular patterns (PAMPs) from bacteria, viruses and fungi, as well as some host molecules [21-24]. It has been suggested that TLRs play a central role in resisting these infections by initiating most of the immune responses that occur during infection [25]. Evidence has shown that TLRs control multiple dendritic cells capable of sensitizing naïve T cells, functions and activates signals that are critically involved in the initiation of adaptive immune responses [26]. Due to their ability to modulate adaptive immunity, TLRs may serve as one of the promising strategic therapeutic targets for diseases related to inappropriate adaptive immune responses, such as sepsis, autoimmune disorders, cancer and allergies [27].

Recently a number of viruses, including a poxvirus, herpesvirus, retrovirus and two paramyxoviruses, have been shown to activate immune cells via TLRs [28-32]. TLR3 is a receptor for double-stranded RNA (dsRNA), through which it transmits signals to activate NF-êB and the interferon -β (IFN-β) promoter and plays an important role in antiviral responses [33-35]. Although the function of type I interferons are most closely associated with their antiviral activities, these cytokines also have diverse effector functions in the development of adaptive immunity. Type I interferons promote the proliferation of memory T cells and prevent T cell apoptosis [36].

Emergent data suggest that the ability of certain individuals to respond properly to TLR ligands may be impaired by single nucleotide polymorphisms (SNPs) located in the *TLR *genes, resulting in an enhanced susceptibility to infectious or inflammatory disease [37-41]. However, whether the genetic variants in TLR3 can alter susceptibility to NPC by affecting the anti-EBV immune responses is unknown. We conducted a case-control study to examine the association between genetic polymorphisms in *TLR3 *and risk of NPC. First, we screened the genomic regions of TLR3 from 24 patients for potential SNPs. Then we genotyped four SNPs in 434 NPC patients and 512 control subjects matched on age, sex and residence, both are of Cantonese origin living in Southern China. Finally, we evaluated the association between those 4 SNPs and the risk of NPC occurrence.

## Methods

### Study population

All subjects were unrelated Cantonese speakers who live in Guangdong Province, Southern China. Patients were recruited from December 2003 to October 2004 with pathologically confirmed diagnosis of NPC at the Cancer Center, Sun Yat-sen University, Guangzhou, China. The average age at NPC diagnosis for all 434 patients who participated in this study was less than 50 years.

The population-based controls were cancer-free individuals and unrelated to the patients. These individuals were selected randomly following a physical examination from a community cancer-screening program for early detection of cancer conducted during the same period as the cases were collected. The selection criteria for control subjects included no individual history of cancer and matched to NPC cases on sex, age (± 5 years), residentiary region and date of blood sample collection. At recruitment, informed consent was obtained from each subject, who was then interviewed to collect detailed information on demographic characteristics. Antibody titers of anti-EBV VCA were also measured on all subjects. This study was approved by the Human Ethics Approval Committee, Cancer Center, Sun Yat-sen University.

### Variation screening

The genomic sequence of *TLR3 *was obtained from the published database of National Center for Biotechnology Information (NCBI) database. The annotations in the database for all known exons, intron-exons boundaries, untranslated regions, and the 5'-flank region (promoter region) of *TLR3 *were used. 12 pairs of primers were designed for the target region using the web-based software Primer 3.0[42]. Each expected fragment size was between 500 and 700 bp. We amplified DNA samples from 24 hospital-based confirmed sporadic NPC patients at Sun Yat-sen University Cancer Center. We then sequenced the PCR products using ABI PRISM Dye Terminator Sequencing Kits and loading the samples onto an ABI^®^377 Automatic Sequencer (Applied Biosystems, Foster City, CA). SNP candidates, identified using the Polyphred/Phredphrap/Consed software package were then confirmed by two independent observers. The SNPs positions and individual genotypes were further confirmed by re-amplification and reverse sequencing. SNPs, with minor allele frequencies above 5%, were entered later for genotype analysis for the case-control study.

### Genotype analysis

Genotype variations were determined by the PCR-based DNA direct sequencing. Genomic DNA was extracted from the peripheral blood samples of all patient cases and control subjects using the DNA zol kit with the protocol from the manufacturer (GibcoBRI, Life Technologies). This genomic DNA sample was amplified by PCR using a GeneAmp9700 PCR System (Applied Biosystems, Foster City CA). The PCR system used a 20-μl reaction mixture containing 20 ng DNA, 0.2 μMol.L^-1 ^of each primer, 200 μMol.L^-1 ^of each deoxynucleotide triphosphate, 1.5 m Mol.L^-1 ^of MgCl_2_, 0.3 units of Taq DNA polymerase with 1 × buffer (Qiagen, Chatsworth, CA). The reaction was conducted under the following conditions: an initial melting step of 5 min at 95°C; followed by 30 cycles of 30s at 94°C, 30s at 60°C, and 45s at 72°C, respectively; and a final elongation step of 5 min at 72°C. Automatic DNA sequencing was performed on an ABI 377 Automatic Sequencer (Applied Biosystems, Foster City, CA) using the direct PCR products of the samples according to the manufacture's protocol. The raw data was collected using ABI PrismTM 377-96 Collection software, and analyzed using Sequencing Analysis software V3.3 on a MAC operating system V9.1, Polyphred/Phredphrap/Consed software package, DNAStar/Taqman software package and CHROMAS. All genotyping was performed blind to case or control status, and blind quality control samples were used to validate genotyping. A random sample of 10% of the cases and controls were sequenced twice by different investigators to confirm genotyping; our reproducibility was 100%.

### Statistical Analysis

For each SNP, deviation from Hardy-Weinberg equilibrium (HWE) in the controls was assessed using the standard *X*^2 ^test. For each SNP, the genotypic frequency differences between cases and controls were tested using conditional logistic regression. All analyses were performed using Statistical Analysis System software (SAS, version 8.0, SAS Institute, Cary, NC). The odds ratios (ORs) and 95 % confidence intervals (CIs) were estimated using logistic regression. All statistical tests were two-sided, and a probability level below 0.05(P < 0.05) was used as the criterion for a significance. Haplotype analyses were conducted using Haplo.stats, which is a score test from generalized linear models (GLM) to test associations between haplotypes and disease under the null hypothesis of no haplotype effect without any assumption about mode of inheritance. This software provides several different global and haplotype-specific tests for association and allows the possibility to include non-genetic covariates. Furthermore, it also allows computation of permutation P values which helps resolve potential problems with sparse data.

## Results

A summary of characteristics of cases and controls is shown in Table 1. No statistical differences were observed between cases and controls in the distribution of age (*p *= 0.6302) and gender (*p *= 0.6415). Table 1 also describes the distribution of VCA-IgA antibody titer category of the cases and controls. The distributions between the two groups were different (P < 0.05). The level of the VCA-IgA antibody titer was positively correlated with the occurrence of NPC, supporting the hypothesis that EBV is strongly associated with NPC.

**Table 1:** Distribution of characteristics of study subjects

**Characteristic**		**Cases (N = 434)**	**Controls (N = 512)**	***p*-value^a^**
Gender, N (%)	Male	322(74.19)	373(72.85)	
	Female	112(25.81)	139(27.15)	0.6415
Age, N(%)	< 30 years	102(23.50)	122(23.83)	
	30–39 years	167(38.48)	186(36.33%)	
	40–49 years	115(26.50)	131(25.59%)	
	> = 50 years	50(11.52)	73(14.26%)	0.6302
VCA_IgA(%)^b^	0 (< 1:10)	28 (6.85)	404(81.65)	
	1 (1:10–1:20)	32(7.82)	70(14.14)	
	2 (1:40)	31 (7.58)	16 (3.23)	< 0.0001
	3 (> = 1:80)	318 (77.75)	5 (1.01 %)	
	No data	25	17	

Note: ^a^*P*-values obtained from chi-square tests. ^b ^Subjects were divided into 4 titer categories: 0, Subjects with VCA-IgA antibody titers less than 1: 10; 1, Subjects with VCA-IgA antibody titers equal to 1: 10 or 1: 20; 2, Subjects with VCA-IgA antibody titers equal to 1: 40; 3, Subjects with VCA-IgA antibody titers more than 1:80. Not all the cases and controls have EBV VCA antibody titers available.

### Selecting SNPs

We examined the total length of TLR3, spanning 6.23 kb. We selected four SNPs, including one SNP (13909C/T, rs3775291 in NCBI bank), which causes amino acid substitutions Leu>Phe, one synonymous (Pro) SNP (13766C/T, N.D.), two SNPs in the noncoding region (829A/C in intron1, N.D., and 9948C/T in intron2, rs5743312). These SNPs with minor allele frequencies above 5%, were used for additional association studies. DNA samples extacted from whole blood in 434 sporadic NPC cases and 512 controls were used for genotyping. Genotypic distributions in the control subjects did not differ significantly from HWE (data not shown).

### Single SNP association

We analyzed the single SNP association with NPC risk separately using conditional logistic regression. The estimated odds ratios (ORs) are provided in Table 2. Chi-squared tests revealed that the variant "829A/C" had a significantly different frequency in cases as compared to controls. The frequency of the allele C for the SNP 829A/C was 14.45% and 10.21% in the cases and controls, respectively (P = 0.0068), and the estimated OR was 1.49 (95%CI: 1.10–2.00). The distributions of the other three SNPs were not different between cases and controls (Table 2).

**Table 2:** Allele frequencies of *TLR3 *SNP and their contributions to the risk for NPC

**Variants**	**Cases**	**Controls**	**P-value**	**OR(95% CI)**
829A/C				
NO. of major allele(%)	681(85.55)	862 (89.79)		
NO. of minor allele(%)	115 (14.45)	98 (10.21)	0.0068	1.49(1.10–2.00)

9948C/T				
NO. of major allele(%)	640 (79.01)	316 (79.00)		
NO. of minor allele(%)	170(20.99)	84 (21.00)	0.9960	1.00(0.74–1.36)

13766C/T				
NO. of major allele(%)	565(71.52)	510(68.73)		
NO. of minor allele(%)	225 (28.48)	232 (31.27)	0.2338	0.88(0.70–1.10)

13909C/T				
NO. of major allele(%)	405 (60.81)	359(62.54)		
NO. of minor allele(%)	261 (39.19)	215(37.46)	0.5318	1.08(0.85–1.36)

### Association with haplotypes

We omitted haplotypes with frequencies less than 0.006, and analyzed the remaining 6 haplotypes with Haplo.stats to evaluate their effects for each haplotype in developing NPC. The results are displayed in Table 3. When adjusted for age, gender and VCA-IgA antibody titers, the calculated NPC risk was significantly reduced among individuals who carried the haplotype "ATCT" (p-value = 0.0054, OR = 0.028, 95% CI (0.002–0.341)), compared to those who carried the most common haplotype "ACCT".

**Table 3:** Haplotype frequencies of the *TLR3 *and effect of environmental factors revealed by Haplo.GLM

**Model**	**Frequencies**		***P*-value^a^**	**OR(95%CI)^a^**
			
	**Cases**	**Controls**		
**Covariates**				
Sex			0.1705	0.530(0.214–1.31)
Age			0. 2019	0.975 (0.938–1.01)
VCA_IgA^b^			< 0.0001	9.91 (6.17–15.9)
**Haplotypes**				
Haplo-1 ATCT	0.0070	0.0151	0.0054	0.028 (0.002–0.341)
Haplo-2 ACTC	0.0797	0.1155	0.0587	0.363 (0.127–1.03)
Haplo-3 CCCC	0.1464	0.1132	0.5554	0.752 (0.292–1.94)
Haplo-4 ATTC	0.1753	0.2027	0.7950	1.10(0.528–2.30)
Haplo-5 ACCT	0.3839	0.3576	Not applicable	Reference
Haplo-6 ACCC	0.1782	0.1955	0.3789	1.40 (0.663–2.95)

Note: ^a^*P*-values and ORs for haplotypes were adjusted by sex, age and VCA-IgA. ^b ^The *P*-values and OR for VCA-IgA was based on "per titer category" as indicated in Table 1.

## Discussion

Nasopharyngeal carcinoma (NPC) is an endemic multi-factorial genetic disease, whereas the disease is rare in the Western world, it occurs with high frequencies in Southern China, Southeast Asia, and among the Greenland Inuit. Genetic susceptibility, EBV infection, environmental risk factors and certain dietary factors are now thought to be associated with the etiology of human NPC. EBV infection has been identified consistently as an important risk factor for NPC, with a dose-response relationship between EBV antibodies and NPC risk [43,44]. A high VCA-IgA antibody titer (> = 1:10) is an index of EBV reactivation. Our results further support the previously reported literature. The positive rate, detected by VCA-IgA antibody titer, is 18.35% in control subjects and 93.15%, respectively in case subjects (Table 1).

*TLR3 *plays an important role in antiviral responses. In this study, we hypothesized that genetic variants in *TLR3 *may be associated with individual susceptibility to NPC. Therefore, we performed a genetic analysis of *TLR3 *sequence variants in 434 NPC cases and 512 controls. We found that the allele C for the SNP 829A/C increased the overall risk of NPC by 49% in the study population (95%CI: 1.10–2.00, P = 0.0068). Because cases and controls were matched in age and gender, the difference between normal control and patients was not likely caused by these factors. Furthermore, the reasonable sample size of this study subjects increases our confidence in interpreting the results.

We also assessed the interaction of these SNPs in NPC risk. When adjusted for age, gender and VCA-IgA antibody titers, the risk was significantly lower in individuals who carried the haplotype "ATCT" (p-value = 0.0054, OR = 0.028; 95% CI (0.002–0.341), compared to those who carried the most common haplotype "ACCT". These results indicate that the SNPs commonly linked to the "ATCT" haplotype are likely to be protective. We recognize that the difference in these two haplotypes is caused by one SNP, 9948C/T.

However, since 9948C/T has no direct association with NPC and some haplotypes with "9948" for example, "ACTC" and "ATTC" did not support any direct protective effect of 9948T, therefore, we conclude that the NPC associated SNPs are unknown but linked to the "ATCT" haplotype and in strong LD with the SNP 829 A/C. Since the SNP 829 is located in an intronic region, it by itself should not possess any direct contribution to the protein function. Therefore the SNP 829 may only be linked to an unknown causative SNP.

Although an observed 1.49-fold increase in NPC risk is modest, this is the magnitude of risk that one would anticipate for a heterogeneous genetic disease. Moreover a similar effect on certain genetic variants has been reported for other diseases that are also related to environmental, genetic variation and viral infection. For example, the etiology of prostate cancer is associated with genetic alterations in environmental carcinogen metabolism, DNA repair and virus infection. Each individually related gene in multiple pathways may alter the risk for prostate cancer, and might contribute only modest risk. In addition, this phenomenon has been observed in other complex diseases, as reported in a recent meta-analysis on the genetic association of complex diseases [45]. In this study, after evaluating 301 published studies that attempted to replicate reported disease-associations for 25 different genes, the authors confirmed the disease-associations for eight of these genes. Interestingly, seven of the eight genes were associated with modest estimated genetic effects (OR between 1.07 and 1.76) in the pooled analyses. It was concluded that there are probably many common variants in the human genome with modest effects on common disease risk. Thus, the observed 49% increase in risk in this study, although modest, are consistent with the hypothesis of common disease/common variants.

## Conclusion

Our study provides evidence of an association between a *TLR3 *sequence variant and NPC risk in the Cantonese population. Although the contribution of the SNPs in *TLR3 *is modest, these sequence variations, together with polymorphisms of other "minor-effect" genes, may define a genetic susceptibility background for NPC, suggesting that SNP 829 may linked to the unknown but important SNPs, and TLR3 gene variation may play an important role in the occurrence of NPC. If our findings are replicated, it will be valuable to further investigate the pathological role of TLR3 polymorphisms in carcinogenesis.

## Competing interests

The author(s) declare that they have no competing interests.

## Authors' contributions

JFH participated in the design of the study and carried out the molecular genetic studies, participated in the sequence alignment and drafted the manuscript. WHJ participated in the design of the study and performed the statistical analysis. QF, XXZ and HDQ participated in the sequence alignment. YYS performed the statistical analysis. YXZ conceived of the study, and participated in its design and coordination. All authors read and approved the final manuscript.

## Pre-publication history

The pre-publication history for this paper can be accessed here:


